# 

**Published:** 1961-01

**Authors:** 


					r
'''' C' ^ S 7
THE
MEDICAL JOURNAL
OF THE
SOUTH-WEST
Incorporating
The Bristol Medico-Chirurgical Journal
January 1961
vol.
76 (i). No. 279 Price 4/-

				

